# Supplementary material for: Human Bone Marrow-Derived Mesenchymal Stromal Cells Reduce the Severity of Experimental Necrotizing Enterocolitis in a Concentration-Dependent Manner
Source: Cells. 2023 Feb 27;12(5):760. doi: 10.3390/cells12050760 (PMC10000931; doi:10.3390/cells12050760)
Supplement: Supplementary file 1 [file cells-12-00760-s001.zip › cells-2182509-supplementary/Table S1.pdf]

Table S1. Term formula milk, human and mouse breast milk composition.

	Term Infant formula	Human Breast Milk	Mouse Breast Milk
Protein (g)	1.5	1.1	9
Fat (g)	3.4	4.2	13.1
Carbohydrates (g)	7.8	7	3
Energy (kcal)	68	72	171
Osmolality (mOsm/kg)	213	~ 300	
Values are calculated on 100mL of milk. g: gram; kcal: kilocalorie; kg: kilogram; mOsm: milliosmole.			
