# Supplementary figures and images for: Human Bone Marrow-Derived Mesenchymal Stromal Cells Reduce the Severity of Experimental Necrotizing Enterocolitis in a Concentration-Dependent Manner
Source: Cells. 2023 Feb 27;12(5):760. doi: 10.3390/cells12050760 (PMC10000931; doi:10.3390/cells12050760)

**A)**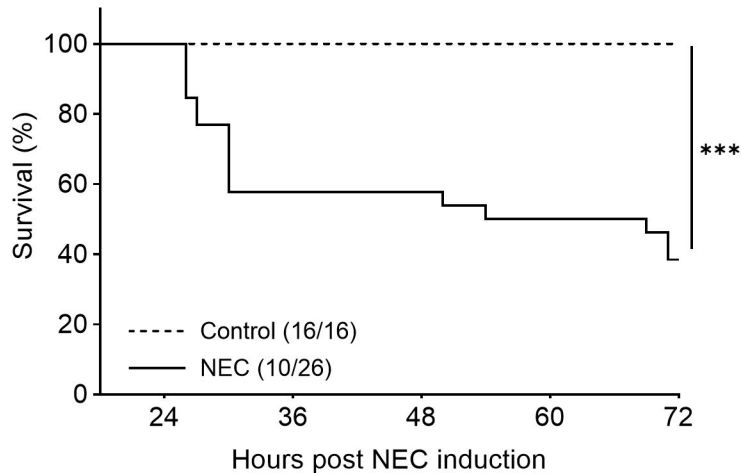**B)**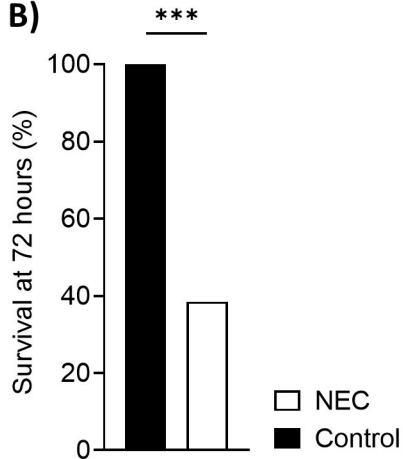

Supplement: Supplementary file 1 [file cells-12-00760-s001.zip › cells-2182509-supplementary/Figure S1.pdf]

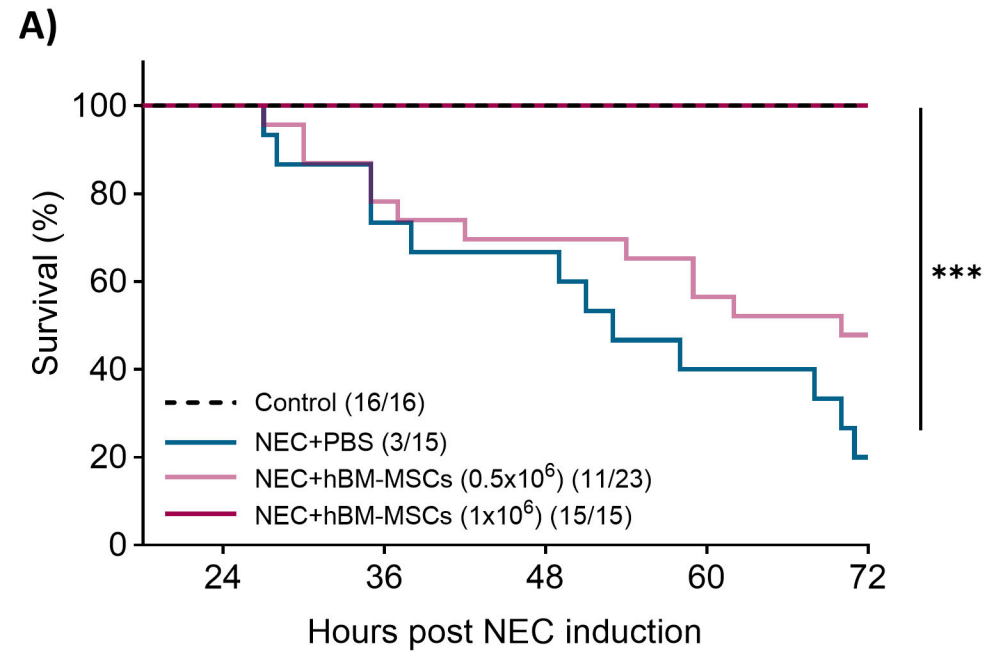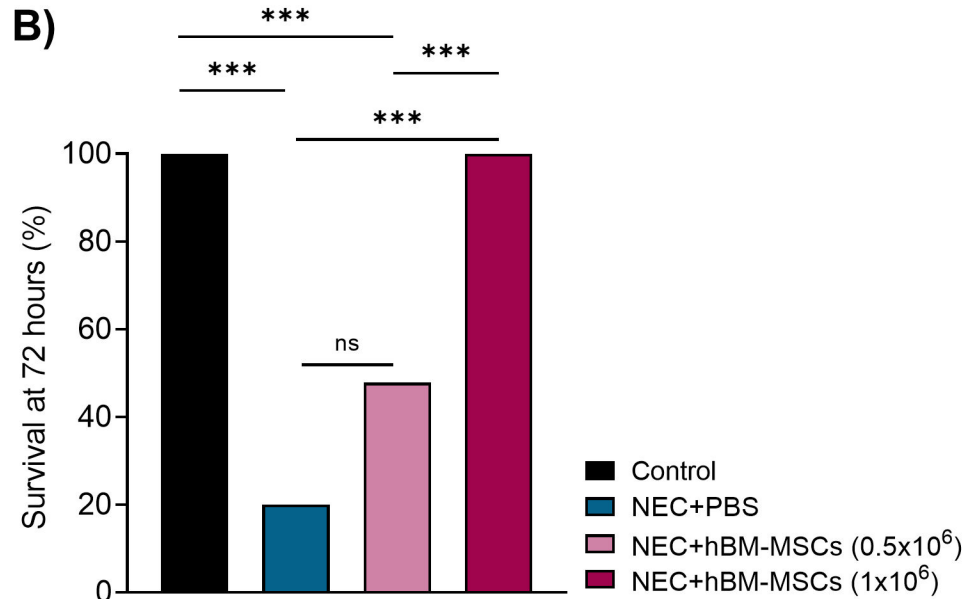

Supplement: Supplementary file 1 [file cells-12-00760-s001.zip › cells-2182509-supplementary/Figure S2.pdf]
